# Radio Frequency Vacuum Drying Study on the Drying Characteristics and Quality of Cistanche Slices and Analysis of Heating Uniformity

**DOI:** 10.3390/foods13172672

**Published:** 2024-08-24

**Authors:** Ao Chen, Fangxin Wan, Guojun Ma, Junmin Ma, Yanrui Xu, Zepeng Zang, Xinyu Ying, Haiwen Jia, Xiaopeng Huang

**Affiliations:** College of Mechanical and Electrical Engineering, Gansu Agricultural University, Lanzhou 730070, China; chena@st.gsau.edu.cn (A.C.); wanfx@gsau.edu.cn (F.W.); magj@gsau.edu.cn (G.M.); majm@gsau.edu.cn (J.M.); xuyr@st.gsau.edu.cn (Y.X.); zangzp@st.gsau.edu.cn (Z.Z.); yingxy@st.gsau.edu.cn (X.Y.); jiahw@st.gsau.edu.cn (H.J.)

**Keywords:** hot air–radio frequency vacuum combined drying, drying uniformity, drying characteristics, quality analysis, microstructure

## Abstract

To fully leverage the advantages of both hot air drying and radio frequency vacuum drying, a segmented combination drying technique was applied to post-harvest *Cistanche*. This new drying method involves using hot air drying in the initial stage to remove the majority of free water, followed by radio frequency vacuum drying in the later stage to remove the remaining small amount of free water and bound water. During the radio frequency vacuum drying (RFV) phase, the effects of temperature (45, 55, and 65 °C), vacuum pressure (0.020, 0.030, and 0.040 MPa), plate spacing (65, 75, and 85 mm), and slice thickness (4, 5, and 6 mm) on the drying characteristics, quality, and microstructure of *Cistanche* slices were investigated. Additionally, infrared thermal imaging technology was used to examine the surface temperature distribution of the material during the drying process. The results showed that compared to radio frequency vacuum drying alone, the hot air–radio frequency combined drying significantly shortened the drying time. Under conditions of lower vacuum pressure (0.020 MPa), plate spacing (65 mm), and higher temperature (65 °C), the drying time was reduced and the drying rate increased. Infrared thermal imaging revealed that in the early stages of hot air–radio frequency vacuum combined drying, the center temperature of *Cistanche* was higher than the edge temperature. As drying progressed, the internal moisture of the material diffused from the inside out, resulting in higher edge temperatures compared to the center and the formation of overheating zones. Compared to natural air drying, the hot air–radio frequency vacuum combined drying effectively preserved the content of active components such as polysaccharides (275.56 mg/g), total phenols (38.62 mg/g), total flavonoids (70.35 mg/g), phenylethanoid glycosides, and iridoids. Scanning electron microscopy observed that this combined drying method reduced surface collapse and cracking of the material. This study provides theoretical references for future drying processes of *Cistanche*.

## 1. Introduction

*Cistanche deserticola is* a tall herbaceous parasitic plant that thrives by parasitizing the roots of the desert tree *Haloxylon ammodendron*. It is widely distributed in Gansu, Ningxia, Inner Mongolia, and Xinjiang in China. *Cistanche* is valued for its ability to tonify essence and blood, dredge intestines, and moisten stools, and has the title of desert ginseng [1]. Moreover, the medicinal material is rich in many phenylethanoid glycosides and iridoids, which possess significant pharmacological activities and can be utilized in the production of antibiotics and anticancer drugs [2]. Nonetheless, *Cistanche* has a high moisture content, making it susceptible to damage during harvesting, transportation, and processing. This susceptibility can lead to issues such as mold, decay, reduced shelf life, diminished quality, and economic losses.

As an important method for preserving agricultural products, drying can effectively reduce the moisture content of the material to inhibit the reproduction of microorganisms, better retain the effective components of the material, and improve its economic value [3]. At present, various drying techniques such as hot air, far-infrared, vacuum freeze, and microwave drying have been employed in research on *Cistanche* slices. However, traditional hot air drying will harden the surface of the material, and high-temperature drying for a long period of time will damage the food color and the natural active ingredients more [4]. Far-infrared drying is a kind of radiative drying by radiating far-infrared rays with a wavelength greater than 4 nm from a heat source, which is converted into thermal energy and absorbed on the surface of the material, but due to the small penetration depth, there are some limitations in the application to the material, and there is a blind spot of irradiation, which is prone to lead to the non-uniform temperature distribution [5]. Vacuum freeze-drying equipment is costly, and the whole drying process is in a continuous low temperature and vacuum state with high energy consumption [6]. Microwave drying has fast heating speed and high energy utilization, but it is prone to cause high surface hardness of the material and expansion and rupture phenomenon [7]. Therefore, Therefore, exploring more suitable drying methods and processes for *Cistanche* is still necessary.

Radio frequency (RF) heating, also known as capacitive medium heating, offers significant advantages such as deep penetration and high efficiency in heat generation. Generally, high-frequency electromagnetic fields with a frequency range of 3 kHz–300 MHz to heat the material [8]. Radio frequency waves can directly interact with materials to generate heat. Under the influence of a high-frequency electromagnetic field, electromagnetic waves interact and couple with the materials, causing polar molecules to undergo frictional reciprocating motion, which produces a thermal effect through ionic migration and dipole rotation [9]. The dielectric properties of agricultural products and food are crucial for RF heating. The higher moisture content of the material results in greater absorption of electromagnetic field energy, thereby accelerating the drying rate [10]. In addition, due to its volume heating effect, RF can heat the material inside and outside, effectively retaining the material’s effective components and improving its economic value. RF technology finds wide application in drying processing, sterilizing [11], insecticidal [12], and thawing [13] of foods and medicinal plants.

RF heating technology still faces challenges in fully leveraging its potential for drying processes. This is mainly due to the irregular shape of the material and the uneven absorption of RF energy, leading to localized heating and the occurrence of glow discharge phenomenon [14]. These factors can result in the formation of charred spots or “coking” during the drying process, which adversely affects the quality of the dried products. Xu et al. studied the drying characteristics and quality of *Lycium barbarum* by RF–hot air combined drying [15]. It was found that the microstructure of *Lycium barbarum* after RF drying was more regular, the color was better, and the effective components of *Lycium barbarum* could be better retained. Liu et al. studied the effect of RF-assisted hot air drying on the drying kinetics and quality of *Zanthoxylum bungeanum* [16]. The results showed that it could better reduce the loss of phenols, flavonoids, and volatile aroma. Ai et al. posited that continuous two-stage hot air combined with RF drying can improve the drying rate and nutritional quality of *Amomum villosum*. They concluded that the moisture content decreased to 40%, which is the best moisture transition point for *Amomum villosum* [17]. Peng et al. found that air jet impingement and hot air-assisted RF mixed drying can shorten the drying time of apple slices and better retain the color, total phenol, and VC content of apple slices [18]. Zhang et al. studied the application of hot air-assisted RF in the second stage of drying mango slices. Firstly, the moisture content of mango slices was reduced to about 40% by hot air drying, and then hot air-assisted RF drying was used to continue drying to below the safe moisture content [19]. These studies have shown that the combined drying method can increase the drying rate of the material, reduce the drying time, and better retain the active ingredients of the material.

Therefore, this study attempts to dry *Cistanche* slices using a hot air–radio frequency vacuum combined (HA-RFV) drying method. The purpose of this study is (1) to evaluate the effects of different (RFV) drying conditions on the drying characteristics, quality, and microstructure of *Cistanche* slices in the second section; (2) the infrared thermal imager was used to explore the temperature distribution and heating uniformity of the material during the drying process. This study can provide technical support for the actual drying production of *Cistanche* slices at a later stage.

## 2. Materials and Methods

### 2.1. RF Drying Equipment

**Figure 1:**
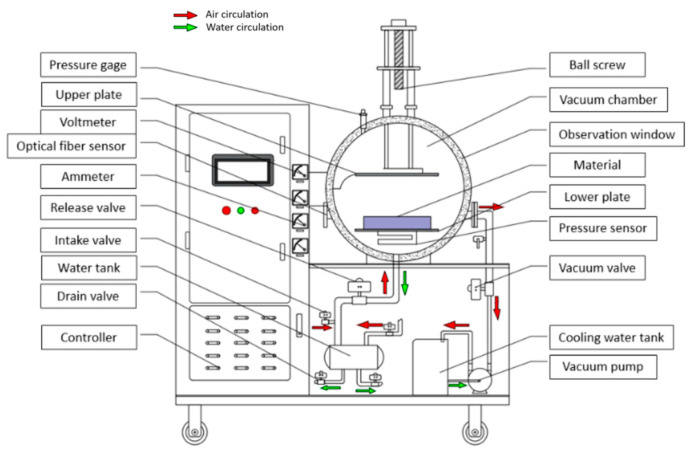
The test device of radio frequency vacuum medium heating.

In this study, the GJS-3-27-JY radio frequency vacuum medium heating test apparatus (Hebei Huashi Jiyuan High Frequency Equipment Co., Ltd., Langfang, China) was used, which is consistent with the equipment used by Xu et al. research [15]. Its schematic diagram is shown in Figure 1. The apparatus mainly consists of a control system, radio frequency heating system, and vacuum system. The high-frequency oscillation power is 3 kW, with a rated frequency of 27.12 MHz, and the ultimate vacuum pressure is −0.09 MPa. The temperature, weight, and vacuum pressure of the material are measured by fiber optic temperature sensors, weighing sensors, and pressure sensors, respectively, and the data are sent in real time to the control panel.

A DHG-9053A desktop blast drying oven (voltage 220 V/50 HZ, input power 1100 W, air velocity 3 m/s, control range: RT + 10~200 °C/RT + 10~250 °C, constant temperature fluctuation ±1 °C, Shanghai Yiheng Scientific Instrument Co., Ltd., Shanghai, China) was used.

### 2.2. Test Materials and Methods

#### 2.2.1. Testing Material

**Figure 2:**
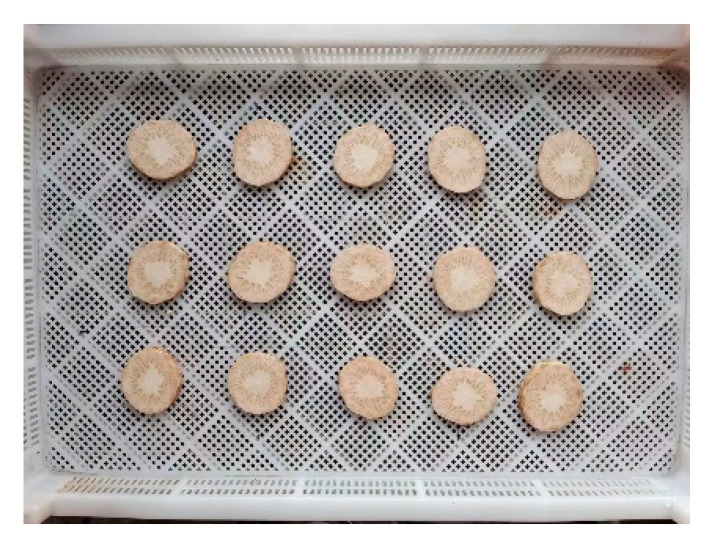
Fresh *Cistanche* slices.

As shown in Figure 2, The fresh *Cistanche* used in this experiment was purchased from Jiuquan (Gansu, China). After being excavated in March, it was vacuum-packed and transported to the laboratory and stored in a constant temperature and humidity box at 4 °C for use, ensuring that the experiment was completed within a month.

Pine echinacoside (standard), salidroside (standard), verbascoside (standard), isoacteoside (standard), calycosin (standard), motherwort glycosides (standard), catalpol (standard), phenol, 1,1-diphenyl-2-picrylhydrazyl (DPPH), catechin, Folin–Ciocalteu, acetonitrile, and gallic acid were analytically pure (simple and pure). Other reagents, such as methanol, were analytically pure, all of which were purchased from Chengdu Pufeide Biotechnology Co., Ltd., Chengdu, China.

#### 2.2.2. Test Method

According to the AOAC (Association of Official Analytical Chemists, no: 934.06,1934) official analysis method, the moisture content (MC) of fresh *Cistanche* was 72.70 ± 0.2% (wet base). Before the test, the materials with no damage on the surface were selected, peeled after cleaning, and sliced after the surface moisture was drained.

**Figure 3:**
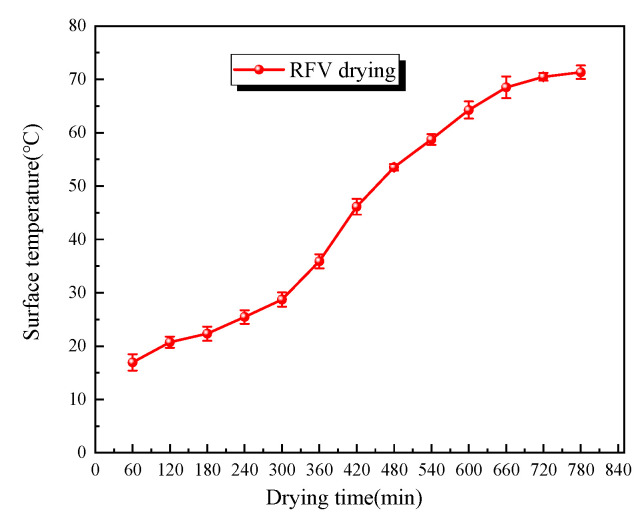
Surface mean temperatures of RFV-dried *Cistanche* slices at different time intervals.

**Figure 4:**
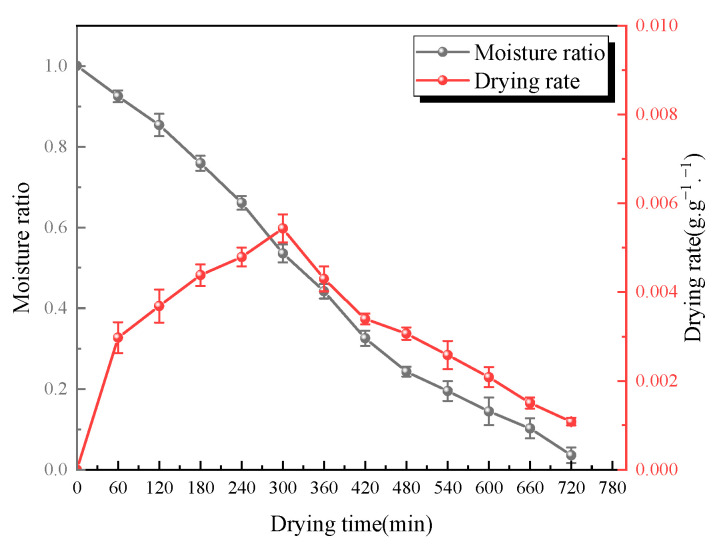
Moisture ratio and drying rate of RFV-dried *Cistanche* slices at different time periods.

In the preliminary experiments, it was found that the drying time of *Cistanche* slices under RFV drying (temperature 55 °C, vacuum pressure 0.030 MPa, plate spacing 75 mm) was approximately 12 h. During the drying process, the surface temperature of the material was recorded every hour using an infrared thermal imager. As shown in Figure 3, the surface temperature of *Cistanche* slices showed little change during the first 5 h of RFV drying, but the surface temperature increased rapidly after 6 h, reaching a maximum of 70 ± 0.2 °C. As shown in Figure 4, the results of the preliminary experiments indicate that the moisture ratio of the material decreased rapidly during the first 5 h of RFV drying, with fast moisture evaporation. After 5 h, the drying rate of the material began to decrease. It was calculated that the moisture ratio of *Cistanche* slices at the 5th hour of RFV drying was 53.58 ± 0.2%; thus, this moisture ratio was determined to be the transition point for combined drying.

In the preliminary experiments, it was found that due to the slow evaporation of moisture in the early stages of RFV drying, the material had prolonged contact with hot and humid air, leading to severe oxidation and surface blackening. Therefore, a combined hot air and radio frequency vacuum drying method was designed to overcome the slow heating drawback of the initial stage of radio frequency vacuum drying. Based on the preliminary experiment results, the optimal hot air drying temperature was determined to be 55 °C.

The hot air–RF vacuum combined drying test steps are as follows:

1. Hot Air Drying: The *Cistanche* slices were first spread flat in the hot air drying oven, and the drying temperature was set to 55 °C to quickly raise the surface temperature of the slices. The weight was recorded every 30 min. It was calculated that after 2 h of hot air drying, the moisture ratio reached the optimal transition point for combined drying (53.58 ± 0.2%). The slices were then immediately transferred to the RFV drying equipment for the second stage of drying;
2. Radio Frequency Vacuum Drying: To determine the optimal parameters for the second stage of RFV drying of *Cistanche* slices, the parameter settings of the RF equipment model and the results of previous experiments were considered. It was found that lower vacuum pressures and thinner slice thicknesses led to uneven heating and edge effects in *Cistanche*. Additionally, too low of plate spacing caused glow discharge within the RF chamber. Therefore, the factor levels for the second stage of RFV drying were selected, as shown in Table 1. The hot air-dried *Cistanche* slices were placed in perforated rectangular plastic trays made of polystyrene, as shown in Figure 2, and then placed in the RFV dryer. The weight was recorded every 30 min, and the surface temperature was captured using an infrared thermal imager until the moisture content of the material decreased to 10 ± 0.2%, at which point the drying process was concluded.

Control group:

Radio frequency vacuum drying: Slices of *Cistanchis* were placed flat on the tray, and the parameters of the radio frequency vacuum drying box were set as follows: temperature 55 °C, vacuum pressure 0.030 MPa, plate spacing of 75 mm. Weighing occurred at intervals of 1 h, and pictures were taken with an infrared thermal imager to record until the moisture content of the material decreased to 10 ± 0.2% and then stopped drying.

Hot air drying: The *Cistanche* slices were laid flat on the tray and put into the hot air drying oven with the temperature of the oven set at 55 °C and air velocity at 3 m/s. Weighing occurred at intervals of 1 h, and pictures were taken with an infrared thermal imager to record until the moisture content of the material dropped to 10 ± 0.2% and then stopped drying.

Natural drying: The *Cistanche* slices were spread on trays and placed in a well-ventilated place at room temperature (25 °C) for natural drying until the moisture content of the material was reduced to (10 ± 0.2%). Then, the drying process was terminated.

To minimize errors, all experiments were repeated three times, and the average value was taken as the experimental result.

### 2.3. Calculation of Drying Characteristic Parameters

#### 2.3.1. Calculation of Dry Base Moisture Content

The dry base moisture content is the ratio of the moisture weight of the wet material to the absolute dry weight of the wet material, as shown in Equation (1):

(1) $$M_{t}=\frac{m_{t}-m_{g}}{m_{g}}\times 100\%$$

Among them, $\;M_{t}$ indicates the dry base moisture content of *Cistanche* at time *t*, %; $m_{t}$ indicates the weight of *Cistanche* at time *t*, g; and $m_{g}$ indicates the weight of dry matter, g ($m_{g}$ = initial material weight − initial material weight × wet base moisture content).

#### 2.3.2. Calculation of Moisture Ratio

The moisture ratio is an important indicator to measure the drying rate of the material in the drying process, as shown in Formula (2) [20]:

(2) $$MR=\frac{M_{t}-M_{e}}{M_{0}-M_{e}}$$

where  MR indicates the moisture ratio of *Cistanche* at t moment; $M_{t}$ indicates the dry base moisture content of *Cistanche* at time *t*, %; $M_{0}$ indicates the initial dry base moisture content of Cistanche, %; and $M_{e}$ indicates the dry base moisture content of *Cistanche* when it is dried to equilibrium, %. Because Me is much smaller than $M_{0}$ and *M_t_*, it can be approximated as 0; therefore, the equation for the moisture ratio of *Cistanche* at different drying times can be simplified as Equation (3) [21].

(3) $$MR=\frac{M_{t}}{M_{0}}$$

#### 2.3.3. Calculation of Drying Rate

The drying rate refers to the amount of water evaporated by the material at different intervals, that is, the change in the moisture content of the material, as expressed in Formula (4) [22]:

(4) $$DR=\frac{M_{t1}-M_{t2}}{t_{2}-t_{1}}$$

Among them, $M_{t1}$, $M_{t2}$ is the dry base moisture content of *Cistanche* at a certain time, %; and $t_{1}$,$\;t_{2}$ is the drying time, min.

### 2.4. Method for Determining the Active Ingredient of Cistanche

#### 2.4.1. Preparation of Polysaccharides, Total Flavonoids, Total Phenols, and Antioxidant Extracts

The dried *Cistanche* slices were ground into powder and filtered with a 0.177 mm sieve, and 0.5 g powder was accurately weighed and immersed in 25 mL of 75% anhydrous ethanol in a plastic centrifuge tube. Subsequently, it was placed in a shaker and oscillated for 48 h at room temperature in the dark (25 °C, 250 r/min). The supernatant was taken after centrifugation for 10 min (20 °C, 3000 r/min) and stored in a 4 °C incubator. The polysaccharides, total flavonoids, total phenols, and antioxidant capacity of the solution were determined by an ultraviolet spectrophotometer [23].

#### 2.4.2. Determination of Polysaccharides

The content of polysaccharides in *Cistanche* slices was determined by the phenol sulfate method, referring to the determination method [24]. Each group of experiments was repeated three times, and the calculation formula is shown in Equation (5):

(5) $$\mathrm{Polysaccharide}\;\mathrm{content}\;\mathrm{mg}/g\cdot \mathrm{DW}=\frac{C\times V_{2}}{V_{1}\times M}$$

$$\mathrm{Standard}\;\mathrm{curve}\;\mathrm{equation}\;Y=0.012X+0.006\left(R^{2}=0.995\right)$$

where C is the concentration of polysaccharides, mg/mL;$\;V_{1}$ is the volume of the aspirated sample solution, mL; $V_{2}$ is the volume of aspirated extract, mL; and  M is the mass of *Cistanche* powder, g.

#### 2.4.3. Determination of Total Flavonoids

The sodium nitrite–aluminum nitrate–sodium hydroxide method was used to determine the content of total flavonoids in *Cistanche* slices, referring to the determination method, and repeated three times in each group of experiments [25]. The calculation formula is shown in Equation (6):

(6) $$\mathrm{Total}\;\mathrm{flavonoid}\;\mathrm{content}\;\mathrm{CEmg}/\mathrm{gDW}=\frac{C\times V_{2}}{V_{1}\times M}$$

$$\mathrm{Standard}\;\mathrm{curve}\;\mathrm{equation}\;Y=0.005X+0.004\;\;\;\;\;\;\;\;\left(R^{2}=0.995\right)$$

Among them, C is the concentration of catechins, mg/mL; $V_{1}$ is the volume of the aspirated sample solution, mL; $V_{2}$ is the volume of aspirated extract, mL; and M is the mass of *Cistanche* powder, g.

#### 2.4.4. Determination of Total Phenols

The content of total phenolic compounds was determined by the forinphenol reagent method, referring to the determination method [25]. Each group of experiments was repeated three times, and the calculation formula is shown in Equation (7):

(7) $$\mathrm{Total}\;\mathrm{phenolic}\;\mathrm{content}\;\mathrm{GAEmg}/\mathrm{gDW}=\frac{C\times V_{2}}{V_{1}\times M}$$

$$\mathrm{Standard}\;\mathrm{curve}\;\mathrm{equation}\;Y=0.036X+0.003\left(R^{2}=0.997\right)$$

Among them, C is the concentration of gallic acid, mg/mL; $V_{1}$ is the volume of the aspirated sample solution, mL; $V_{2}$ is the volume of the aspirated extract, mL; and cap Mis is the mass of Stanche powder, g.

#### 2.4.5. Determination of Antioxidant Properties (DPPH Method)

The total antioxidant capacity of organic active substances was determined by the DPPH method [26], and the calculation formula is shown in Equation (8):

(8) $$\mathrm{Inhibition}\;\mathrm{rate}\;=\frac{\left(A_{0}-A\right)}{A_{0}}\times 100\%$$

Among them, A is the absorbance value of the aspiration extract; and $\;A_{0}$ is the absorbance without extract.

#### 2.4.6. Determination of Phenhexyl Glycosides and Cyclic Ether Terpene Components in Cistanche (HPLC Method)

A total of 1 g of *Cistanche* powder was weighed, and 70% methanol was added to a constant volume of 25 mL. It stood for 30 min, was subjected to ultrasonic treatment (70 °C, 40 min, 700 W, 45 KHz), and then centrifuged (3000 r/min, 4 °C, 10 min). The supernatant was extracted and stored in a 4 °C incubator for later use.

The reference substances of azalpin, motherwort, salidroside, echinaceaside, chrysmin, verbascoside, and isobolicoside were weighed to 3 mg, and 3 mL of chromatographic methanol was added. A reference substance with a concentration of 1 mg/mL was made and diluted at 0.5 times to a concentration of 0.125 mg/mL, 0.0625 mg/mL, 0.03125 mg/mL, 0.015625 mg/mL, 0.0078125 mg/mL, and 0.00390625 mg/mL of mixed standard control solution.

The High-Performance Liquid Phased Instrument (HPLC) method was used to refer to the determination method [27]. The chromatographic conditions were as follows: column: Agilent Eclipse XDB-B-C-18 (250 mm × 4.6 mm, 5 μm); column temperature: 30 °C; volume flow rate 1.0 mL/min; injection volume: 1 μL; gradient elute: mobile phase water (A)–acetonitrile (B); 0~6 min, 90~10%; 6~10 min, 80~20%; 10~15 min, 75~25%; 15~20 min, 70~30%; 20~24 min, 65~35%; 24~26 min, 35~65%; 26~28 min, 55~45%; 28~30 min, 90~10%; detection wavelength: 205 mm.

### 2.5. Determination of Chromatic Aberration Value

The surface color of *Cistanche* slices was measured by a precision colorimeter (CS-210, Zhengzhou North-South Instrument Co., Ltd., Zhengzhou, China). The results were expressed as $L^{*}$, $a^{*},$ and $b^{*}$ values. According to the formula [28], the total color difference (ΔE) (9), chroma (10), and hue angle (11) are as follows:

(9) $$\Delta E=\sqrt{{\left(L-L^{*}\right)}^{2}+{\left(a-a^{*}\right)}^{2}+{\left(b-b^{*}\right)}^{2}}$$

(10) $$Chroma=\sqrt{\left({a_{t}}^{2}+{b_{t}}^{2}\right)}$$

(11) $$Hue\;Angle=tan^{-1}\left(\frac{b_{t}}{a_{t}}\right)$$

Among them, ΔE indicates the magnitude of the total color difference of Cistanche; L represents the luminance value of the dried *Cistnache* slices; $L^{*}$ indicates the initial brightness value of fresh *Cistanche* slices; a represents the red-green value of the slice of *Cistanche* to be measured; $\;a^{*}$ indicates the initial red-green value of fresh *Cistanche* slices; b represents the yellow-blue value of the sliced *Cistanche* under test; and $b^{*}$ indicates the yellow-blue value of fresh *Cistanche* slices.

### 2.6. Rehydration Ratio

The degree to which dried products can return to their original fresh state after rehydration is one of the important indicators to measure the quality of dried products [29]. *Cistanche* slices of 5 g under different drying conditions were taken and immersed in 300 mL distilled water at 30 °C. Every hour, the samples were dried with filter paper, and the weight of each sample was recorded. The experiment was repeated three times to obtain the average [30]. The calculation formula is expressed as in Equation (12):

(12) $$R_{f}=\frac{G_{f}}{G_{g}}$$

where $R_{f}$ denotes the rehydration rate; $G_{f}$ represents the mass of *Cistanche* slices after rehydration, g; and $G_{g}$ represents the mass of Cistanche slices before rehydration, g.

### 2.7. Microstructure

The dried *Cistanche* slices were longitudinally cut into small pieces of 3 mm × 3 mm × 2 mm and fixed to the SEM stage with conductive tape. After 60 s of gold spraying treatment by a sputtering instrument, the scanning electron microscope was set to accelerate the voltage of 5.0 kV, the spot size was 50 μm of the 3rd hole, the aperture of the objective lens was 4 μm, the working distance was 12.5 mm, and the surface structure was observed by a magnification of 1400×.

### 2.8. Statistical Analysis

To ensure the reliability of the experimental data, all the analyses in this experiment were repeated three times, and the average value was taken for analysis. Excel 2016 was used to calculate the dry basis moisture content, moisture ratio, and drying rate of *Cistanche* slices. Origin 2018 was used for data fitting and graphics rendering. Analysis of variance (ANOVA) was performed using SPSS 27.0 software (*p* < 0.05).

## 3. Results and Discussion

### 3.1. Drying Characterization of Radio Frequency Vacuum-Dried Cistanche Slices in the Second Segment

#### 3.1.1. Effect of Temperature on Drying Characteristics of *Cistanche*

**Figure 5:**
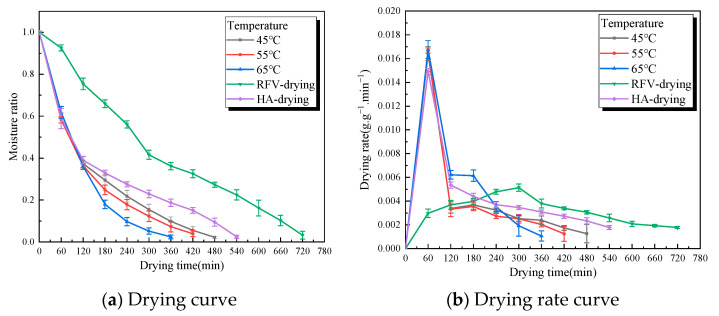
The green line is the characteristic curve of RFV drying of *Cistanche* at a temperature of 55 °C, a vacuum pressure of 0.030 MPa, and a pole-plate spacing of 75 mm. The black, red, and blue lines are the characteristic curves of HA-RFV drying of *Cistanche* at different temperatures (in which the 0–120 min overlapping line part of the drying condition is hot air at 55 °C). The purple line is the drying characteristic curve of *Cistanche* hot air (55 °C).

When the plate spacing was 75 mm, the vacuum pressure was 0.030 MPa. The slice thickness was 5 mm, and the *Cistanche* slices were dried under different RFV temperatures; the moisture ratio and drying rate curves are shown in Figure 5. It can be seen from Figure 5 that the higher the drying temperature, the faster the drying rate, and the shorter the time to dry to the safe moisture content of *Cistanche*. Compared to non-hot air combined drying, the drying time decreased by 33.3%, 41.6%, and 50.0% at 45 °C, 55 °C, and 65 °C, respectively. This shows that under the condition of other conditions unchanged, with the increase in temperature, the surface temperature of the material increases, which leads to the rapid evaporation of free water, the gradient difference between internal and external water formation increases, the diffusion and evaporation of internal water accelerates, and the heat transfer efficiency improves [31]. At higher temperatures, the evaporation rate of water increases, leading to a faster relative drying rate. However, this can cause severe shrinkage of the material. Materials undergo surface hardening and localized charring occurred under high-temperature conditions, reducing the permeability of the material’s interior. This is consistent with the conclusion drawn [32].

#### 3.1.2. Effect of Plate Spacing on Drying Characteristics of Cistanche

**Figure 6:**
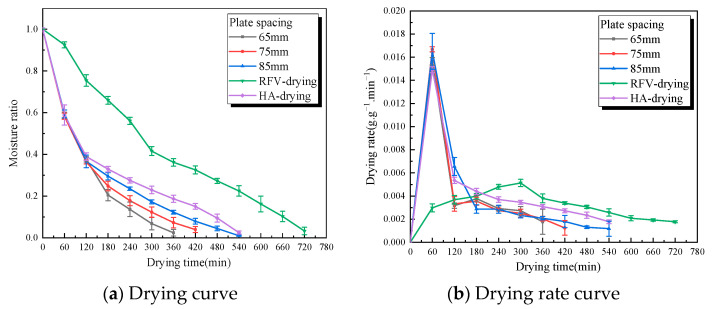
The green line is the characteristic curve of RFV drying of *Cistanche* at a temperature of 55 °C, a vacuum pressure of 0.030 MPa, and a pole-plate spacing of 75 mm. The black, red, and blue lines are the HA-RFV drying characteristic curves of *Cistanche* under different spacings of pole plates (in which the 0–120 min overlapping line part of the drying condition is hot air at 55 °C). The purple line is the drying characteristic curve of *Cistanche* hot air (55 °C).

The moisture ratio and drying rate of RFV drying of *Cistanche* slices under different plate spacings are shown in Figure 6 when the temperature was 55 °C, the vacuum pressure was 0.030 MPa, and the slice thickness was 5 mm. It can be seen from the figure that the drying time of *Cistanche* slices increased with the increase in plate spacing. Compared to non-hot air combined drying, the drying time decreased by 50.0%, 41.6%, and 25.0% when the plate spacing gap was set at 65 mm, 75 mm, and 85 mm, respectively, similar to the research results of Peng et al. [18]. The reduction in the plate spacing has a positive effect on shortening the drying time and increasing the drying rate, which is consistent with the variation in the RF load current with the plate spacing [33]. The RF electric field strength increases with the decrease in the plate spacing, so the smaller the plate spacing, the higher the RF field strength, the more RF energy the material absorbs, resulting in too high a surface temperature of the material, accelerating the evaporation and overflow of water, and increasing its drying rate. However, when the plate spacing is too low, the dielectric properties of the material will affect the electric field distribution, which may lead to the corner effect of energy concentration. In addition, the irregular shape of the material will also cause the uneven distribution of the magnetic field energy, resulting in the carbonization of the heated material [34,35].

#### 3.1.3. Effect of Vacuum on Drying Characteristics of Cistanche

**Figure 7:**
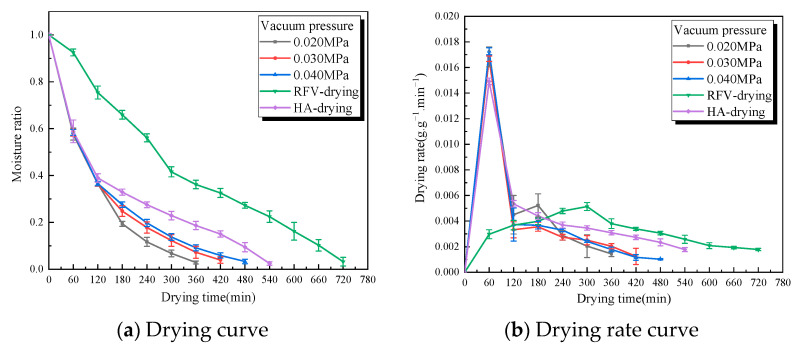
The green line is the characteristic curve of RFV drying of *Cistanche* at a temperature of 55 °C, a vacuum pressure of 0.030 MPa, and a pole-plate spacing of 75 mm. The black, red, and blue lines are the characteristic curves of *Cistanche* under different HA-RFV degrees (in which the 0–120 min overlapping line part of the drying condition is hot air at 55 °C). The purple line is the drying characteristic curve of *Cistanche* hot air (55 °C).

The moisture ratio and drying rate of RFV drying of *Cistanche* slices under different vacuum pressures are shown in Figure 7, when the temperature was 55 °C, the plate spacing was 75 mm, and the slice thickness was 5 mm. With the increase in vacuum pressure, the drying rate of *Cistanche* slices showed a downward trend. Compared to non-hot air combined drying, the drying time decreased by 50.0%, 41.6%, and 33.3% at 0.020 MPa, 0.030 MPa, and 0.040 MPa, respectively. This may be because, on the one hand, the increase in vacuum pressure increases the pressure difference between the inside and outside of the material, increases the friction and collision frequency of the bipolar particles inside the material, and increases the gradient of the temperature difference between the inside and outside, thus accelerating the water diffusion efficiency and shortening the drying time. On the other hand, the increase in vacuum pressure inside the cavity will lead to a decrease in the boiling point of water molecules, which is more conducive to the evaporation of water. Under the combined action of the two, the drying time gradually shortened with the increase in vacuum pressure. When the vacuum pressure is 0.020 MPa, the boiling point of water in *Cistanche* slices is the lowest, and the water inside and outside the sample can rise to the boiling point in a shorter time, effectively reducing the drying time and increasing the drying rate. However, if the vacuum pressure is too low, it usually causes glow discharge in the cabin, which can easily cause local gelatinization of materials and equipment ignition. Local overheating will bring irreversible damage to *Cistanche* slices [36].

#### 3.1.4. Effect of Slice Thickness on Drying Characteristics of Cistanche

**Figure 8:**
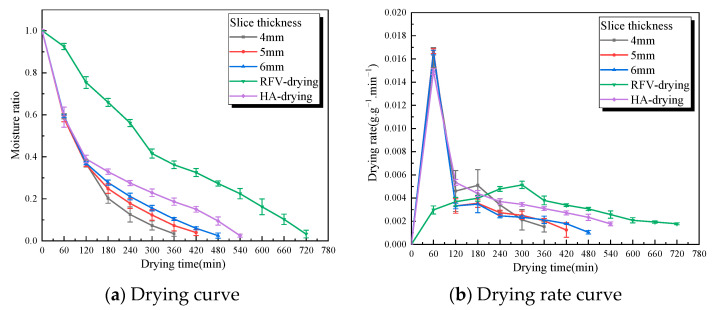
The green line is the characteristic curve of RFV drying of *Cistanche* at a temperature of 55 °C, a vacuum pressure of 0.030 MPa, and a pole-plate spacing of 75 mm. The black, red, and blue lines show the HA-RFV drying characteristic curve of *Cistanche* under different thicknesses of slices (in which the 0–120 min overlapping line part of the drying condition is hot air at 55 °C). The purple line is the drying characteristic curve of *Cistanche* hot air (55 °C).

The moisture ratio and drying rate of RFV drying of *Cistanche* slices under different slice thicknesses are shown in Figure 8, when the temperature was 55 °C, the plate spacing was 75 mm, and the vacuum pressure was 0.030 MPa. The drying time increased by 25% when the thickness of *Cistanche* slices was increased from 4 mm to 6 mm, and the drying rate decreased with the increase in slice thickness, which is similar to the findings [37]. When the thickness of kiwifruit slices increased from 4 mm to 12 mm, the total time of microwave vacuum drying of kiwifruit increased by nearly 300%. This may be due to the strong penetration ability of RF, which can quickly heat the material [38]. The increase in material thickness can lead to the increase in diffusion path and distance of water inside the material, as well as the increase in internal resistance. However, in RF drying, due to the loss of “ion migration” and “dipole rotation”, the friction between molecules causes a large amount of heat inside the sample. The material’s internal temperature gradient and pressure gradient can effectively promote the migration of internal water and the evaporation of surface water [39]. If the thickness of the slice is too thin, it will cause the material to shrink. The strong penetration ability will make the polar molecules inside the material move rapidly, and the edge effect will lead to the carbonization of the material [40].

### 3.2. Computer Vision and Uniformity Analysis

**Figure 9:**
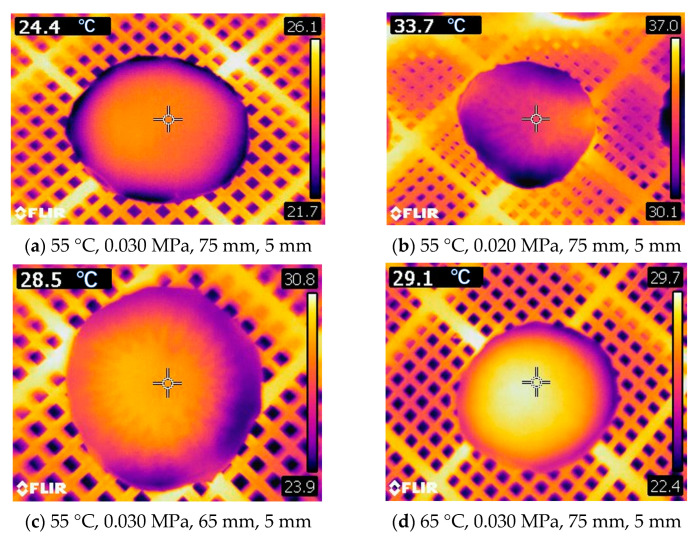
Infrared thermogram of *Cistanche* slices dried under HA-RFV drying conditions for the 3rd hour (i.e., surface temperature distribution of *Cistanche* slices 1 h after transferring from HA to RFV drying).

To investigate the impact of RFV drying parameters on the heating uniformity of *Cistanche* slices, infrared thermal imaging was utilized to obtain images under experimental conditions (55 °C, 0.030 MPa, 75 mm, 5 mm) for both the intermediate group and the group with faster drying rates. Specifically, the conditions for the intermediate group were vacuum pressure (0.020 MPa), plate spacing (65 mm), and temperature (65 °C) during RFV drying. The infrared thermal images under these conditions are presented in Figure 9. From Figure 9b–d, it can be observed that under the RFV drying conditions of lower vacuum pressure, plate spacing, and higher temperature, the temperature distribution of *Cistanche* slices exhibited significant non-uniformity. The electric field between the parallel plates in the RF cavity is uniformly distributed. However, due to differences in the shape and dielectric properties of the material, the absorption of RF energy by the material is uneven, leading to edge effects [41].

**Figure 10:**
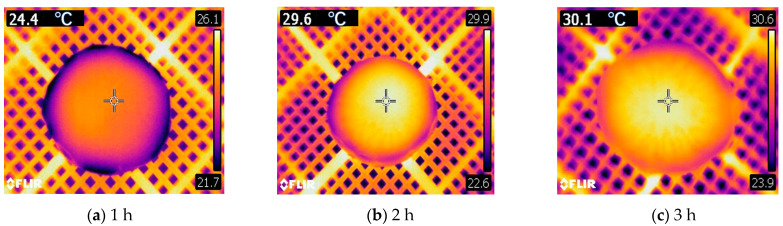

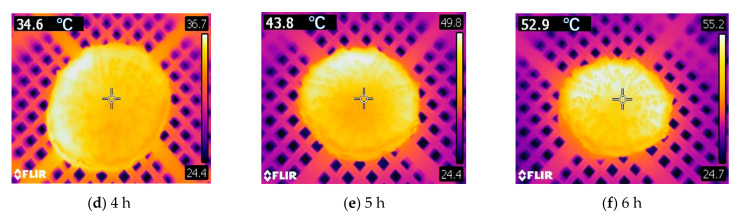
Infrared thermography of *Cistanchis* slices under the conditions of HA-RFV drying (55 °C, 0.030 MPa, 75 mm, 5 mm) for different time periods.

The infrared thermography of the surface temperature of *Cistanche* slices at different time periods of HA-RFV drying is shown in Figure 10. The surface temperature of *Cistanche* slices gradually increased with the prolongation of drying time. In the early stage of drying, the central temperature of the material was higher than the edge temperature. This is mainly attributed to the volumetric heating method employed in RFV drying, where the high-frequency electric field current directly interacts with the material to generate heat, promoting the outward migration of moisture within the material. However, this method can also lead to the formation of overheated regions within the material, which is detrimental to the preservation of heat-sensitive nutrients. As the drying time increased, the temperature at the material’s edge significantly exceeded the internal temperature. This may be due to the uneven distribution of inherent moisture within the material, resulting in uneven temperature distribution during drying [40]. The heating method of RFV drying causes moisture within the material to trend toward outward diffusion, concentrating moisture at the material’s edges. Consequently, the high humidity at the material’s edge absorbs excessive RF energy, resulting in overheated regions at the material’s edges and uneven temperature distribution during drying. Continuous drying gradually raises the surface temperature of *Cistanche* slices. In the later stages of drying, the edges of *Cistanche* slices appear brighter, which is also the area most prone to ignition, carbonization, and “thermal runaway” phenomena. This phenomenon is a crucial cause of decreased quality of *Cistanche* slices due to uneven heating during RF heating [42].

From the infrared thermal imaging maps presented in Figure 9 and Figure 10, it can be observed that during RF heating, *Cistanche* slices exhibit an uneven temperature distribution pattern. This may be attributed to the “edge effect” generated by RF heating, where the dielectric properties of the air inside the cavity and the plastic plates holding the material differ, leading to changes in the electromagnetic field distribution. This difference tends to accumulate at the edges of the material, resulting in uneven heating. The edges of the material absorb more RF energy, causing them to reach higher temperatures than the center [43].

### 3.3. Quality Analysis of Cistanche Slices by Radio Frequency Vacuum Drying Parameters in the Second Segment

#### 3.3.1. Effects of Different RFV Drying Conditions on Polysaccharides Content

**Figure 11:**
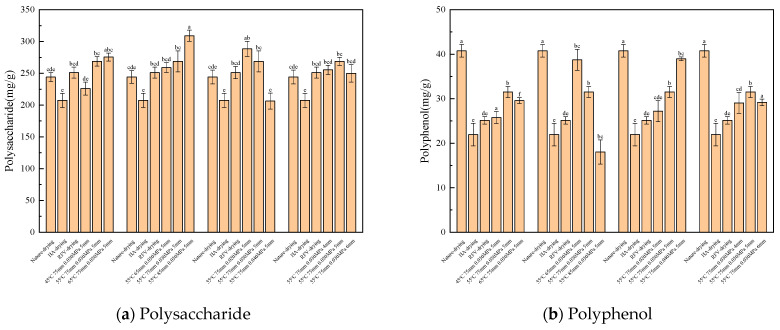

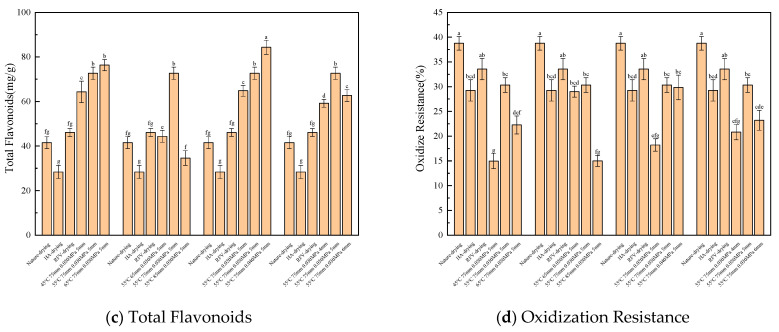
Effects of polysaccharides (**a**), polyphenols (**b**), total flavonoids (**c**), and oxidization resistance (**d**) of *Cistanche* slices under different drying conditions. Note: A different lowercase letter after each column indicates a significant difference (*p* < 0.05).

The polysaccharide content under different RFV drying conditions is shown in Figure 11a. the polysaccharide content with a plate height of 85 mm is the highest (309.08 mg/g), which is significantly higher than that under other conditions (*p* < 0.05). The polysaccharide content of dried products increased with plate height and vacuum pressure. This may be because in the process of dielectric heating, the higher the absolute vacuum pressure is, the greater the internal pressure of the material is, and the starch decomposition in the material is relatively weak so that the polysaccharide content can be better retained. The polysaccharide content at different drying temperatures was 225.96 mg/g (45 °C) < 268.65 mg/g (55 °C) < 275.56 mg/g (65 °C). With the increase in temperature, the drying rate becomes faster, and there is a negative correlation between the content of polysaccharides and the drying time; that is, the longer the drying time, the more serious the degradation of polysaccharides [44].

#### 3.3.2. Effects of Different RFV Drying Conditions on Polyphenols in *Cistanche* Slices

Total phenolic compounds have the effects of anti-oxidation, anti-aging, promoting gastrointestinal digestion, reducing blood fat, and increasing body resistance. The content of total phenols under different drying conditions is shown in Figure 11b. The content of phenols in the control group (natural drying) was the highest (40.55 mg/g), which was significantly higher than that under other conditions (*p* < 0.05). This may be because phenols are chemically unstable substances, and too high a temperature will accelerate the reaction rate of oxidation and thermal degradation [45]. The results showed that the combined HA-RFV drying method could retain polyphenols more effectively than the HA and RFV drying methods. Under the HA-RFV drying test conditions, the polyphenol content in the *Cistanche* slices was affected by temperature, vacuum pressure, and plate spacing. With the increase in slice thickness and drying temperature, the content of phenols in *Cistanche* slices increased first and then decreased. When the slice thickness was 5 mm and the temperature was 55 °C, the content of phenols was the highest (31.5 mg/g). With the increase in plate spacing, phenolic compounds decreased gradually. This is because the drying time under this condition is the shortest, the polyphenol content is 38.62 mg/g, and the drying time is the longest when the plate spacing is 85 mm. The polyphenol content is 18.03 mg/g, and the phenolic substances are oxidized and thermally degraded considerably. With the increase in vacuum pressure, phenols also gradually decreased. The total phenol content under different drying conditions was significantly lower than that of natural drying, which may be due to the continuous drying process that makes the material absorb a large amount of thermal energy. The high temperature of *Cistanche* slices resulted in severe thermal degradation of the oxidizing enzymes and polyphenol oxidizing enzymes inside, leading to a decrease in the total phenol content [31].

#### 3.3.3. Effects of Different RFV Drying Conditions on Total Flavonoids in *Cistanche* Slices

Flavonoids can regulate body function, enhance the body’s resistance to disease, induce tumor cell apoptosis, and promote metabolism. The flavonoid content under different drying conditions is shown in Figure 11c. The total flavonoid content in the control group (natural drying) was the lowest (39.78 mg/g), which was significantly lower than other conditions (*p* < 0.05). When other conditions remain unchanged, the total flavonoids in *Cistanche* slices gradually increase with the increase in temperature. This is because the increase in temperature will lead to violent movement of intercellular molecules, accelerate the decomposition of components, and release more flavonoids. With the increase in the height of the RFV drying plate, the flavonoids increased and then decreased. This is because when the plate height is 65 mm, the total flavonoid content is 41.60 mg/g. Under this condition, the electromagnetic field intensity is larger, and the electromagnetic energy absorbed by the material is greater, so the structure of the flavonoids is destroyed. When the height of the plate increased to 85 mm, the total flavonoid content was 30.75 mg/g, and the drying time under this condition increased by 33.3%. When the slice thickness was 5 mm, the total flavonoid content was the highest (70.35 mg/g). At this time, the surface structure of the material was relatively intact, and more total flavonoids of *Cistanche* slices could be retained, which is consistent with the results of [5]. It was found that the changing trend of total flavonoid content was similar to that of total phenols content, which may be because total phenols and total flavonoids were heat-sensitive components, and they had synergistic effects.

#### 3.3.4. Effects of Different RFV Drying Conditions on the Oxidation Resistance of *Cistanche* Slices

DPPH assay was used to study the antioxidant activity of dried *Cistanche* slices. DPPH is often used to evaluate the ability of materials to scavenge free radicals. The antioxidant activity of *Cistanche* slices under different drying conditions is shown in Figure 11d. The antioxidant capacity of the material after natural drying is the strongest (30.33%), which is significantly higher than other conditions of combined segmented drying (*p* < 0.05). Studies have shown that the antioxidant activity of the material is related to the content of total phenols and flavonoids in the material. An appropriate temperature increase can improve the active substance’s antioxidant capacity. However, if the temperature is too high, the active substance will react too fast, reducing its ability to scavenge free radicals to a certain extent and showing weak antioxidant activity. Also, at high temperatures, the degradation of active components of total phenolic substances and total flavonoids was inhibited, and their antioxidant activity was also destroyed, resulting in lower antioxidant activity than in other groups [40]. The antioxidant activity after RFV drying was significantly lower than that of the control group. This may be due to the cell damage caused by high-intensity electromagnetic fields, which caused the oxidative degradation of glycosides, phenols, and flavonoids in the material, showing weak antioxidant capacity.

#### 3.3.5. Effects of Different RFV Drying Conditions on Phenylethanol Glycosides and Cyclic Ether Terpenes in *Cistanche* Slices

**Figure 12:**
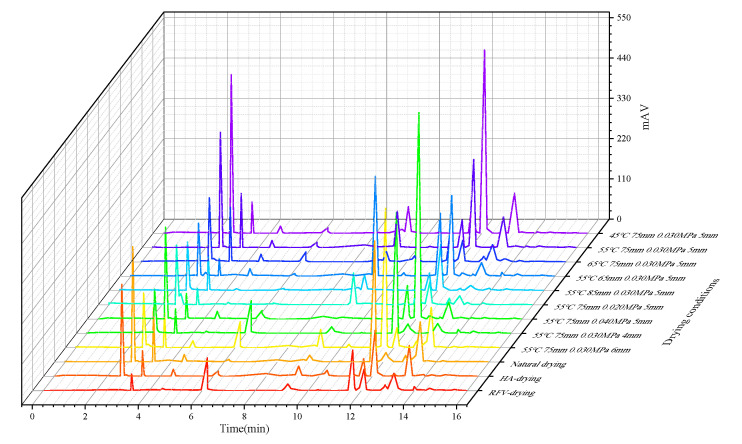
HPLC chromatogram of phenylethanol glycosides and cyclic ether terpene contents of *Cistanche* slices under different drying conditions.

Phenylethanoid glycosides and iridoids are the main natural active ingredients of *Cistanche*. Among them, the content of echinacoside and verbascoside in phenylethanoid glycosides is the highest, including catalpol, leonuride, salidroside, crocin, isoverbascoside, etc., which are the main components with improving effect, regulating immune function, scavenging free radicals, and anti-aging properties [46]. The contents of phenylethanoid glycosides and iridoids in *Cistanche* slices under different drying conditions and natural drying are shown in Figure 12 and Table 2. High-frequency electromagnetic fields generated by RF significantly increased the content of echinacoside and verbascoside (*p* < 0.05), which indicated that RFV drying was beneficial for retaining the heat-sensitive nutrients of the material. When the drying temperature was 65 °C, the contents of echinacoside (0.27 ± 0.004 mg/mL) and verbascoside (0.23 ± 0.03 mg/mL) were the lowest. This may be because phenolic hydroxyl groups and glycosidic bonds are more sensitive to temperature. High-temperature heating destroys its molecular structure and transforms its stable state into an unsaturated or highly reactive state, leading to decreased component content [47]. This is similar to the results of [5]. With the increase in drying time, the natural active ingredients decreased, which may be due to the long drying time and the greater contact between the material and the hot and humid air, so that the effective active ingredients were decomposed under the catalysis of the enzyme.

#### 3.3.6. Effects of Different RFV Drying Conditions on the Color and Rehydration Ratio of *Cistanche*

Color is also an important index in evaluating the drying quality of materials. The browning reaction was observed by measuring the color difference of the sample. In the drying process, the Maillard reaction is the main factor affecting the browning of dried products [48]. As shown in Table 3, the lower the color difference (ΔE), the smaller the color change after drying, and vice versa. After drying, the value of the hue angle of the material decreased from 90.02 ± 1.05 to 71.14 ± 0.49, which may be due to the degradation of the pigment caused by the drying heating process, and the material’s color became darker. The $a^{*}$ value of dried *Cistanche* slices was significantly higher than that of fresh samples (1.64 ± 0.20), indicating that RFV drying can improve the brightness of the samples. This is consistent with the results of [15] study that the dried products after RFV drying of *Lycium barbarum* are brighter and have the smallest ΔE. With the increase in temperature, the color difference (ΔE) of *Cistanche* slices was 17.94 ± 0.66 (45 °C) < 15.11 ± 0.61 (55 °C) < 13.26 ± 0.54 (65 °C), showing a decreasing trend. With the increase in plate spacing, the color difference (ΔE) decreased from 21.46 ± 0.65 to 14.12 ± 0.05, which may be due to the decrease in the probability of Maillard reaction with the increase in material drying rate [49].

The rehydration ratio is used to evaluate the structural characteristics of dried materials. The higher the rehydration ratio is, the looser the material’s internal structure after drying is, and the better the internal pores are [50]. As shown in Table 3, the vacuum pressure during RFV drying has more obvious damage to the structure of *Cistanche*. The rehydration ratio of the samples under different vacuum pressures decreased with the increase in vacuum pressure, and the vacuum pressure was 0.020 MPa, showing weak rehydration. Moreover, the plate spacing was 65 mm, and the lowest value of the rehydration ratio was 2.03 ± 0.01, which is significantly lower than other drying conditions (*p* < 0.05). This may be due to the high-frequency electric field intensity, the penetration force of the electric field being relatively large, and the damage to the internal structure of the material being stronger.

#### 3.3.7. Effects on the Microstructure of Cistanche Slices under Different RFV Drying *Conditions*

**Figure 13:**
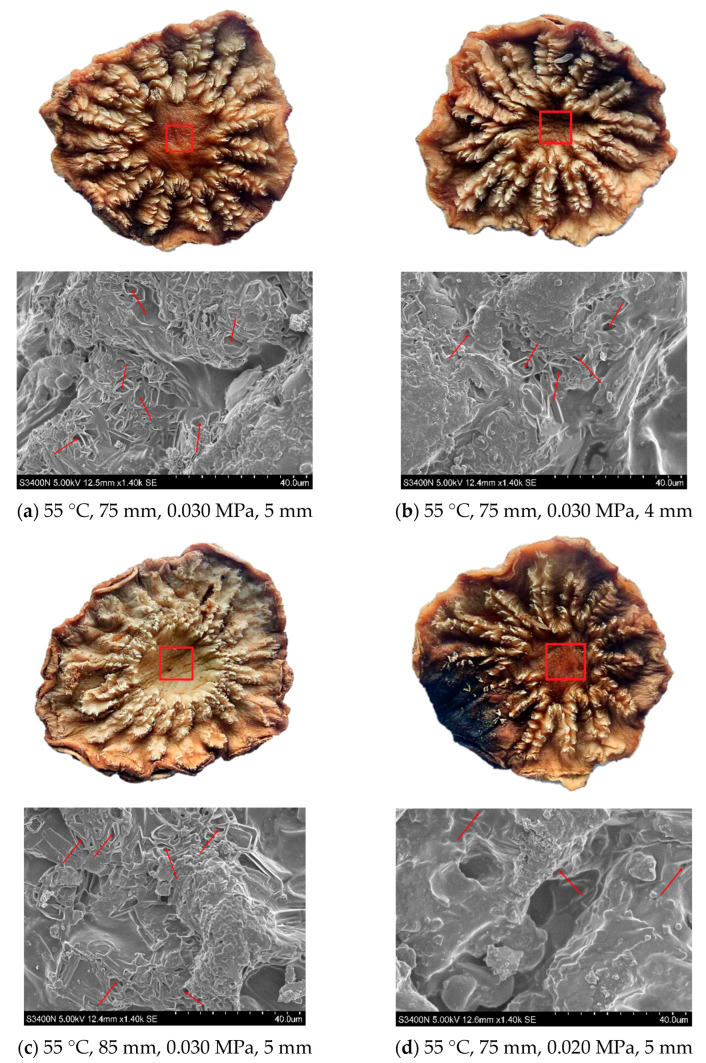
Sample plot and scanning electron microscope diagram of *Cistanche* sections under different HA-RFV drying conditions.

The slices and microstructure of *Cistanche* under different RFV drying conditions are shown in Figure 13, The red boxes on the material indicate the parts observed by the scanning microscope, and the red arrows pointing to the positions indicate the pore structures on the surface of the material. After drying, the material’s surface appears to collapse and gully, which may be caused by cell contraction. The drying process causes the cell wall to rupture, damaging the water exchange channel [51]. The RFV drying conditions were as follows: the temperature was 55 °C, the plate spacing was 75 mm, the vacuum pressure was 0.030 MPa, and the slice thickness was 5 mm. The sample structure and surface pore structure retained the best, the color of the sample after drying was uniform, and the surface structure remained more complete. When the thickness of the slice is 4 mm, the material can better absorb heat. The high temperature makes the material shrink considerably, resulting in a hardening crust on the surface of the sample and a local scorch phenomenon. When the plate spacing is 85 mm, the drying time is the longest. Because the material is in a high-temperature and -humidity environment for a long time, it may lead to slow evaporation of material moisture, and the texture has greater toughness than other groups. When the vacuum pressure increased to 0.020 MPa, the pore size of *Cistanche* slices expanded, and the surface color became black. The surface structure of the material appeared fractured and gully, and the damage to the material’s structure was the most serious. Therefore, the rehydration after drying under this condition was the worst [52], which was consistent with the previous conclusion of the rehydration ratio.

## 4. Conclusions

This experiment investigated the effects of different HA-RFV drying conditions on the drying characteristics, heating uniformity, quality, and microstructure of *Cistanche* slices. The results showed that the segmented drying of HA-RFV could shorten the drying time. The higher the temperature, the lower the vacuum pressure and plate spacing during the RFV drying stage, and the thinner the slice thickness, the shorter the drying time and the faster the drying rate. This could better reduce the phenomenon of glow discharge and carbonization at the edge of the material during RFV drying, avoiding “thermal runaway” caused by continuous RF heating. Under the conditions of RFV drying at 55 °C, a vacuum pressure of 0.030 MPa, a plate spacing of 75 mm, and a slice thickness of 5 mm, the polysaccharide content (268.68 mg/g), total flavonoids (70.35 mg/g), polyphenols (31.51 mg/g), antioxidant activity (36%), cynarin (0.42 ± 0.02 mg/mL), and verbascoside (0.71 ± 0.08 mg/mL) in *Cistanche* slices could be better preserved. The dried products had uniform color, bright color (ΔE = 15.11 ± 0.61), good rehydration (3.049 ± 0.091), and more intact surface pore structure. The infrared thermal imaging showed that in the early stage of drying, due to the volumetric heating method of RFV drying, the direct action of high-frequency electric field current on the material generated heat, promoting the outward migration of moisture in the material, causing the center temperature of the material to be higher than the edge temperature. With the increase in drying time, the moisture inside the material tended to diffuse from the inside to the outside, and moisture gathered at the edge of the material, leading to excessive absorption of RF energy at the edge surface of the material, causing overheating at the edge of the material, and the edge temperature of the material was higher than the center temperature. This paper briefly explored the changes in surface temperature distribution, providing a theoretical basis for subsequent studies on the mass and heat transfer processes of RFV drying of *Cistanche* slices.

## Figures and Tables

**Table 1:** One-way experimental design for the second segment of RFV-dried *Cistanche* slices.

Test Serial Number	Temperature (°C)	Vacuum Pressure (MPa)	Plate Spacing (mm)	Slice Thickness (mm)
1	45	0.030	75	5
2	55	0.030	75	5
3	65	0.030	75	5
4	55	0.020	75	5
5	55	0.040	75	5
6	55	0.030	65	5
7	55	0.030	85	5
8	55	0.030	75	4
9	55	0.030	75	6

**Table 2:** Main component contents of phenylethanol glycosides and cyclic ether terpenes in *Cistanche* slices under different drying conditions.

Drying Conditions	Phenylethanoid Glycoside (mg/mL)					Iridoids (mg/mL)	
	Echinacoside	Salidroside	Jinshi Sericin	Verbascoside	Isoacteoside	Catalpol	Leonurus Japonicus Glycoside
45 °C 75 mm 0.030 MPa 5 mm	$0.93\pm 0.06^{\;c}$	$0.24\pm 0.05^{\;e}$	$0.69\pm 0.05^{\;c}$	$3.35\pm 0.04^{\;b}$	$1.25\pm 0.14^{\;a}$	$27.37\pm 2.63^{\;a}$	$14.38\pm 2.71^{\;e}$
55 °C 75 mm 0.030 MPa 5 mm	$0.47\pm 0.05^{\;\mathrm{cd}}$	$0.31\pm 0.07^{\;d}$	$0.51\pm 0.11^{e\;}$	$7.44\pm 0.41^{\;a}$	$0.45\pm 0.07^{\;b}$	$19.85\pm 1.07^{\;b}$	$20.21\pm 2.03^{\;b}$
65 °C 75 mm 0.030 MPa 5 mm	$0.27\pm 0.04^{\;d}$	$0.43\pm 0.12^{\;b}$	$0.69\pm 0.16^{\;c}$	$0.19\pm 0.04^{\;g}$	$0.36\pm 0.03^{\;\mathrm{de}}$	$10.93\pm 1.09^{\;\mathrm{de}}$	$18.31\pm 1.47^{\;c}$
55 °C 65 mm 0.030 MPa 5 mm	$2.97\pm 0.01^{\;a}$	$0.21\pm 0.01^{\;\mathrm{ef}}$	$1.03\pm 0.09^{\;b}$	$3.08\pm 0.17^{\;\mathrm{bc}}$	$0.39\pm 0.08^{\;\mathrm{cd}}$	$9.06\pm 0.54^{\;\mathrm{ef}}$	$7.85\pm 0.65^{\;\mathrm{fg}}$
55 °C 85 mm 0.030 MPa 5 mm	$0.28\pm 0.06^{\;d}$	$0.18\pm 0.01^{\;\mathrm{fg}}$	$0.36\pm 0.01^{\;g}$	$1.32\pm 0.04^{\;d}$	$0.18\pm 0.02^{\;\mathrm{fg}}$	$8.26\pm 0.03^{\;f}$	$11.23\pm 1.23^{\;\mathrm{ef}}$
55 °C 75 mm 0.020 MPa 5 mm	$1.63\pm 0.15^{\;b}$	$0.14\pm 0.07^{\;g}$	$0.56\pm 0.05^{\;\mathrm{de}}$	$0.92\pm 0.08^{\;\mathrm{de}}$	$0.27\pm 0.03^{\;e}$	$10.25\pm 0.31^{\;e}$	$5.45\pm 0.01^{\;g}$
55 °C 75 mm 0.040 MPa 5 mm	$0.37\pm 0.08^{\;d}$	$0.39\pm 0.02^{\;\mathrm{bc}}$	$0.59\pm 0.04^{\;d}$	$7.77\pm 0.21^{\;a}$	$0.13\pm 0.04^{\;\mathrm{fg}}$	$15.81\pm 0.03^{\;\mathrm{cd}}$	$9.67\pm 0.62^{\;f}$
55 °C 75 mm 0.030 MPa 4 mm	$0.23\pm 0.05^{\;d}$	$1.16\pm 0.09^{\;a}$	$1.88\pm 0.03^{\;a}$	$0.14\pm 0.04^{\;g}$	$0.07\pm 0.01^{\;g}$	$7.75\pm 0.43^{\;\mathrm{fg}}$	$14.34\pm 0.21^{\;e}$
55 °C 75 mm 0.030 MPa 6 mm	$0.33\pm 0.12^{\;d}$	$0.95\pm 0.02^{\;\mathrm{ab}}$	$2.15\pm 0.06^{\;a}$	$0.23\pm 0.01^{\;f}$	$0.08\pm 0.02^{\;g}$	$9.33\pm 0.07^{\;\mathrm{ef}}$	$14.87\pm 1.74^{\;e}$
Natural drying	$2.67\pm 0.51^{\;b}$	$0.21\pm 0.03^{\;\mathrm{ef}}$	$1.71\pm 0.05^{\;b}$	$5.53\pm 0.02^{\;b}$	$0.41\pm 0.07^{\;\mathrm{bc}}$	$14.71\pm 0.13^{\;d}$	$24.55\pm 1.45^{\;a}$
HA drying	$0.49\pm 0.05^{\;\mathrm{cd}}$	$0.24\pm 0.01^{\;e}$	$0.38\pm 0.02^{\;g}$	$0.56\pm 0.06^{\;f}$	$0.18\pm 0.02^{\;f}$	$6.45\pm 0.06^{\;g}$	$14.06\pm 0.32^{\;e}$
RFV drying	$0.23\pm 0.04^{\;d}$	$0.36\pm 0.06^{\;c}$	$0.43\pm 0.06^{\;f}$	$3.24\pm 0.04^{\;c}$	$0.23\pm 0.05^{\;\mathrm{ef}}$	$16.24\pm 0.41^{\;c}$	$16.63\pm 1.03^{\;d}$

Note: A different lowercase letter after each column indicates a significant difference (*p* < 0.05).

**Table 3:** Color and rehydration ratio parameters of *Cistanche* slices under different drying conditions.

Drying Conditions	Color				Rehydration Ratio
	$L^{*}$	$a^{*}$	$b^{*}$	ΔE	R (%)
Fresh sample	$78.22\pm 0.01^{\;a}$	$1.64\pm 0.20^{\;g}$	$28.21\pm 0.87^{\;\mathrm{ab}}$		
45 °C 75 mm 0.030 MPa 5 mm	$63.71\pm 0.67^{\;d}$	$4.65\pm 0.05^{\;e}$	$19.43\pm 0.24^{\;\mathrm{bc}}$	$17.94\pm 0.66^{\;b}$	$2.65\pm 0.01^{\;b}$
55 °C 75 mm 0.030 MPa 5 mm	$66.51\pm 0.91^{\;\mathrm{bc}}$	$6.06\pm 0.15^{\;a}$	$21.59\pm 0.37^{\;a}$	$15.11\pm 0.61^{\;\mathrm{def}}$	$3.05\pm 0.09^{\;a}$
65 °C 75 mm 0.030 MPa 5 mm	$68.59\pm 0.78^{\;b}$	$5.12\pm 0.21^{\;c}$	$21.40\pm 0.16^{\;\mathrm{bc}}$	$13.26\pm 0.54^{\;\mathrm{fg}}$	$2.64\pm 0.02^{\;b}$
55 °C 65 mm 0.030 MPa 5 mm	$60.70\pm 0.60^{\;e}$	$6.22\pm 0.24^{\;a}$	$18.20\pm 0.49^{\;\mathrm{bc}}$	$21.46\pm 0.65^{\;a}$	$2.03\pm 0.01^{\;g}$
55 °C 85 mm 0.030 MPa 5 mm	$60.53\pm 0.26^{\;e}$	$5.53\pm 0.20^{\;b}$	$17.68\pm 0.29^{\;c}$	$14.12\pm 0.05^{\;\mathrm{efg}}$	$2.47\pm 0.05^{\;cd}$
55 °C 75 mm 0.020 MPa 5 mm	$65.85\pm 0.46^{\;\mathrm{cd}}$	$4.58\pm 0.55^{\;\mathrm{ef}}$	$20.90\pm 0.28^{\;\mathrm{bc}}$	$15.44\pm 0.64^{\;\mathrm{cde}}$	$2.17\pm 0.11^{\;\mathrm{fg}}$
55 °C 75 mm 0.040 MPa 5 mm	$64.59\pm 1.08^{\;\mathrm{cd}}$	$4.81\pm 0.14^{\;\mathrm{de}}$	$19.81\pm 0.33^{\;\mathrm{bc}}$	$17.09\pm 0.99^{\;\mathrm{bc}}$	$2.39\pm 0.04^{\;\mathrm{de}}$
55 °C 75 mm 0.030 MPa 4 mm	$66.39\pm 1.13^{\;\mathrm{bc}}$	$4.26\pm 0.28^{\;f}$	$20.13\pm 0.56^{\;\mathrm{bc}}$	$15.34\pm 1.13^{c\;\mathrm{de}}$	$2.56\pm 0.04^{\;\mathrm{bc}}$
55 °C 75 mm 0.030 MPa 6 mm	$67.04\pm 1.41^{\;\mathrm{bc}}$	$6.08\pm 0.17^{\;a}$	$22.27\pm 0.46^{\;\mathrm{abc}}$	$14.38\pm 1.37^{\;\mathrm{egf}}$	$2.31\pm 0.02^{\;\mathrm{ef}}$
Natural drying	$59.23\pm 1.34^{\;e}$	$3.24\pm 0.11^{\;\mathrm{gf}}$	$24.04\pm 0.42^{\;\mathrm{abc}}$	$16.53\pm 0.31^{\;\mathrm{bcd}}$	$1.76\pm 0.01^{\;h}$
HA drying	$63.52\pm 0.63^{\;d}$	$4.75\pm 0.32^{\;\mathrm{de}}$	$25.31\pm 0.74^{\;\mathrm{abc}}$	$12.47\pm 0.56^{\;g}$	$2.08\pm 0.14^{\;g}$
RFV drying	$60.37\pm 1.27^{\;e}$	$5.04\pm 0.54^{\;\mathrm{cd}}$	$20.08\pm 1.04^{\;\mathrm{bc}}$	$13.74\pm 0.28^{\;\mathrm{egf}}$	$2.24\pm 0.27^{\;\mathrm{ef}}$

Note: A different lowercase letter after each column indicates a significant difference (*p* < 0.05).

## Data Availability

The original contributions presented in the study are included in the article, further inquiries can be directed to the corresponding author.
